# Axicabtagene ciloleucel treatment is more effective in primary mediastinal large B-cell lymphomas than in diffuse large B-cell lymphomas: the Italian CART-SIE study

**DOI:** 10.1038/s41375-024-02213-x

**Published:** 2024-03-08

**Authors:** Annalisa Chiappella, Beatrice Casadei, Patrizia Chiusolo, Alice Di Rocco, Silva Ljevar, Martina Magni, Piera Angelillo, Anna Maria Barbui, Ilaria Cutini, Anna Dodero, Francesca Bonifazi, Maria Chiara Tisi, Stefania Bramanti, Maurizio Musso, Mirko Farina, Massimo Martino, Mattia Novo, Giovanni Grillo, Francesca Patriarca, Giulia Zacchi, Mauro Krampera, Martina Pennisi, Eugenio Galli, Maurizio Martelli, Andrés J. M. Ferreri, Silvia Ferrari, Riccardo Saccardi, Anisa Bermema, Anna Guidetti, Rosalba Miceli, Pier Luigi Zinzani, Paolo Corradini

**Affiliations:** 1 Division of Hematology and Stem Cell Transplantation, Fondazione IRCCS Istituto Nazionale dei Tumori, Milano, Italy; 2 IRCCS Azienda Ospedaliero-Universitaria di Bologna, Istituto di Ematologia “Seràgnoli”, Bologna, Italy; 3 Department of Diagnostica per Immagini, Radioterapia Oncologica ed Ematologia, Fondazione Policlinico Universitario A. Gemelli IRCCS, Roma, Italy; 4 Hematology Section, Department of Translational and Precision Medicine, “Sapienza” University of Rome, Roma, Italy; 5 Unit of Biostatistics for Clinical Research, Department of Data Science, Fondazione IRCCS Istituto Nazionale dei Tumori, Milano, Italy; 6 Lymphoma Unit, IRCCS San Raffaele Scientific Institute, Milano, Italy; 7 Department of Oncology and Hematology, Azienda Socio-Sanitaria Territoriale Papa Giovanni XXIII, Bergamo, Italy; 8 SOD Terapie Cellulari e Medicina Trasfusionale, Azienda Ospedaliero-Universitaria Careggi, Firenze, Italy; 9 Hematology Unit, San Bortolo Hospital, A.U.L.S.S. 8 “Berica”, Vicenza, Italy; 10 Department of Oncology/Hematology, IRCCS Humanitas Research Hospital, Rozzano, Milano, Italy; 11 UOC di oncoematologia e TMO “La Maddalena”, Palermo, Italy; 12 Unit of Blood Disease and Bone Marrow Transplantation, and Unit of Hematology, University of Brescia, ASST Spedali Civili di Brescia, Brescia, Italy; 13 Stem Cell Transplantation and Cellular Therapies Unit (CTMO), Department of Hemato-Oncology and Radiotherapy, Grande Ospedale Metropolitano “Bianchi-Melacrino-Morelli”, Reggio Calabria, Italy; 14 Division of Hematology, Città della Salute e della Scienza Hospital and University, Torino, Italy; 15 Dipartimento di Ematologia e trapianto di midollo, ASST Grande Ospedale Metropolitano Niguarda, Milano, Italy; 16 Clinica Ematologica ed Unità Terapie Cellulari, Azienda Sanitaria Universitaria Friuli Centrale, Dipartimento di Area Medica, Università di Udine, Udine, Italy; 17 SCDU Ematologia AO SS Antonio e Biagio e Cesare Arrigo ed Università del Piemonte Orientale, Alessandria, Italy; 18 UOC di Ematologia e Centro Trapianto di Midollo Osseo – Azienda Ospedaliera Universitaria Integrata Verona Policlinico G.B. Rossi, Verona, Italy; 19 Chair of Hematology, University of Milano, Milano, Italy; 20 Dipartimento di Scienze Mediche e Chirurgiche, Università di Bologna, Bologna, Italy

**Keywords:** B-cell lymphoma, Cancer immunotherapy

## Abstract

Axicabtagene ciloleucel showed efficacy for relapsed/refractory large B-cell lymphomas (LBCL), including primary mediastinal B-cell lymphomas (PMBCL); however, only few PMBCLs were reported. Aim was to evaluate efficacy and safety of axicabtagene ciloleucel in patients with PMBCL compared to those with other LBCL, enrolled in the Italian prospective observational CART-SIE study. PMBCLs (*n* = 70) were younger, with higher percentage of bulky and refractory disease, compared to other LBCLs (*n* = 190). Median follow-up time for infused patients was 12.17 months (IQR 5.53,22.73). The overall (complete + partial) response rate (ORR,CR + PR) after bridging was 41% for PMBCL and 28% for other LBCL, *p* = 0.0102. Thirty days ORR was 78% (53/68) with 50% (34) CR in PMBCL, and 75% (141/187) with 53% (100) CR in other LBCL, *p* = 0.5457. Ninety days ORR was 69% (45/65) with 65% (42) CR in PMBCL, and 54% (87/162) with 47% (76) CR in other LBCL; progressive disease was 21% in PMBCL and 45% in other LBCL, *p* = 0.0336. Twelve months progression-free survival was 62% (95% CI: 51–75) in PMBCL versus 48% (95% CI: 41–57) in other LBCL, *p* = 0.0386. Twelve months overall survival was 86% (95% CI: 78–95) in PMBCL versus 71% (95% CI: 64–79) in other LBCL, *p* = 0.0034. All grade cytokine release syndrome was 88% (228/260); all grade neurotoxicity was 34% (88/260), with 6% of fatal events in PMBCL. Non-relapse mortality was 3%. In conclusion, PMBCLs achieved significantly better response and survival rates than other LBCLs.

## Introduction

Primary mediastinal large B-cell lymphomas (PMBCL) represent 6–10% of aggressive large B-cell lymphomas (LBCL), ~80–85% of them can be cured with first line chemoimmunotherapy [1].

Before the recent immunotherapies, the standard approach for relapsed/refractory (R/R) diseases was still based on chemotherapy and rituximab followed by autologous stem cell transplantation (autoSCT) in chemo-sensitive patients, with a 5-year Overall Survival (OS) ranging from 57% to 71% in those who were able to completed the therapeutic program [2–4]. When looking at the patients with refractory disease status at autoSCT, the 3-year estimate for OS was 22–41%, suggesting that the refractory patients did worse and need to be treated with different approaches [2, 4].

In PMBCL, a relevant biological insight was the demonstration of the amplification of 9p24.1, resulting in overexpression of programmed death ligand-1 and programmed death ligand-2 (PD-L1/L2) [5]. These genetic features represented the rationale for testing programmed death 1 blockade with check-point inhibitors (CPIs) in R/R disease. Pembrolizumab demonstrated very interesting survival outcomes in the KEYNOTE-170 study [6, 7]. Based on the expression of CD30 in PMBCL, the combination of Nivolumab plus Brentuximab-Vedotin was also tested, with even better survival outcomes in the CheckMate 436 study [8, 9].

In diffuse large B-cell lymphomas (DLBCL), the outcome of refractory disease is unsatisfactory, as reported in the SCHOLAR-1 study: in 636 refractory DLBCL patients, the ORR was 26%, with 7% CR, and the median OS was 6.3 months, with 1-year OS of 28% [10].

Recently, the chimeric antigen receptor (CAR) T-cell therapies targeting the CD19 antigen became the novel standard for salvage treatment of R/R LBCL. After failure of at least two lines of therapy, axicabtagene ciloleucel (axi-cel), tisagenlecleucel (tisa-cel) and lisocabtagene maraleucel (liso-cel) demonstrated impressive efficacy in the ZUMA-1, JULIET and TRANSCEND trials respectively [11–13]. In the ZUMA-1 trial, axi-cel demonstrated an ORR of 83% in 111 LBCL patients, with 58% CR, and an estimated 2-year and 5-year OS of 50.5% (95% CI: 40.2–59.7) and 42.6% (95% CI: 32.8–51.9), respectively [14, 15]. However, only eight patients with PMBCL were included in this pivotal trial; at a median follow-up of 27 months, ongoing responses were maintained in five of eight cases [14]. There are only two real-world retrospective experiences, one reporting the outcome of 33 patients treated with axi-cel in United States, with ORR 78% and 69% CR, and 2-year PFS and OS 64% (95% CI: 49–84) and 78% (95% CI: 64–96), respectively [16]; the second one, is a retrospective sub-analysis of the German registry, reporting the outcome of 13 PMBCL with 2‐year PFS and OS of 54% and 75% [17].

Thus, it is still unclear whether PMBCL have a different survival outcome compared to other LBCL. In Italy, we are conducting a prospective multicenter observational study (CART-SIE) to evaluate efficacy and toxicity of CAR-T in lymphomas; the present analysis evaluated the outcome of PMBCL and other LBCL patients treated with axi-cel.

## Patients and methods

### Study design and participants

CART-SIE is an ongoing multicenter prospective observational study, coordinated by the “Fondazione IRCCS Istituto Nazionale dei Tumori”, Milano, Italy and conducted in collaboration with the Italian Society of Hematology in 21 Italian hematological centers approved by regulatory authorities for CAR T-cell therapy administration.

All patients eligible to CAR T-cell therapy in accordance with “Agenzia Italiana del Farmaco” (AIFA, Italian drug agency) criteria (the detailed list of eligibility criteria in accordance with AIFA is provided in the supplementary materials) were consecutively enrolled.

The study was conducted in accordance with the Declaration of Helsinki and good clinical practice guidelines. Ethical approval was obtained by institutional review boards at each site (INT 180/19, approval number 431/DG, 2019). All participants provided written informed consent. AIFA was notified on August 27, 2019.

Eligible patients were R/R patients affected by LBCL, including DLBCL [DLBCL not otherwise specified (DLBCL-NOS), DLBCL arising from transformed follicular lymphoma (tFL)], high-grade B-cell lymphoma (HGBCL) and PMBCL, after at least two treatment lines, with an Eastern Cooperative Oncology Group Performance Status (ECOG PS) 0-1, treated with CAR T-cell therapy. A detailed list of inclusion and exclusion criteria is provided in the supplementary materials.

All patients underwent the planned lymphodepletion chemotherapy with 30 mg/ms fludarabine and 500 mg/ms cyclophosphamide on days −5, −4 and −3, as per clinical practice; all patients received the planned infusion of axi-cel. We compared the outcome of PMBCL versus other LBCL, in term of ORR, Duration of Response (DoR), OS and PFS, cytokine release syndrome (CRS), immune-effector cell associated neurotoxicity syndrome (ICANS) and non-relapse mortality (NRM).

### Treatment and clinical assessment

All patients were treated with in-label axi-cel per institutional decision, outside clinical trials. Clinical response assessment was assessed by Lugano criteria at each center, without centralized review [18].

### Statistical methods

Efficacy measures were calculated as follows:

- ORR: the percentage of responding patients was estimated as the number of patients with CR or with partial response (PR) divided by the total number of patients assessable at each specific timepoint. Patients not assessable for response for any reason were considered as non-responding in calculations. The 95% exact binomial confidence intervals of the response percentage were also estimated.
- DoR: for patients who responded to treatment, DoR was measured as the interval between the response achievement and the date of progression or death, whichever occurred first, with censoring at the date of the latest follow-up in alive patients without progression. DoR curves were estimated with the Kaplan Meier method.
- OS: time was measured as the interval between the date of CAR T-cell infusion and the date of death for all causes, with censoring at the date of the latest follow-up in alive patients. OS curves were estimated with the Kaplan Meier method.
- PFS: time was measured as the interval between the CAR T-cell infusion and the date of progression disease (PD), or death, whichever occurred first, with censoring at the date of the latest follow-up in alive patients without progression. PFS curves were estimated with the Kaplan Meier method.
  Between groups comparisons of Kaplan-Meier curves were performed using the log rank test, and comparison at specific timepoints in remission were calculated using the chi-square test proposed by Klein et al. [19].

Safety was evaluated as follows:

- NRM after CAR T-cell therapy was measured as the interval between the date of CAR T-cell infusion and the date of non-relapse death, with censoring at the date of the latest follow-up in alive patients without relapse. NRM cumulative incidence curves were estimated regarding disease recurrence as competing event, and between groups comparisons were performed using the Gray test [20].
- CRS and ICANS were graded according to modified Lee criteria and the American Society for Transplantation and Cellular Therapy (ASCTC) criteria [21]. Descriptive statistics were used to summarize these data. Hematological and non-hematological toxicities were graded according to the Common Terminology Criteria for Adverse Events (CTCAE) Version 5. (Published: November 27. US Department of Health and Human Services, National Institutes of Health, National Cancer Institute). Descriptive statistics were used to summarize these data [22].

The binary association between continuous and categorical variables was assessed using the Mann–Whitney–Wilcoxon or the Kruskal Wallis tests, as appropriate; the Fisher-Freeman-Halton test was used when testing association between two categorical variables [23].

## Results

### Patients characteristics

**Fig. 1:**
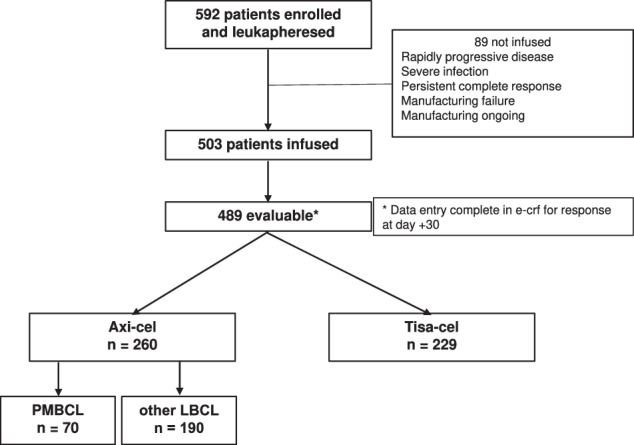
Patient flow: March 2019–July 2023. e-crf electronic Case Report Form, axi-cel axicabtagene ciloleucel, tisa-cel tisagenlecleucel, PMBCL primary mediastinal B-cell lymphoma, other LBCL large B-cell lymphoma other than primary mediastinal B-cell lymphoma.

Since March 2019 to June 2023, 592 R/R PMBCL and other LBCL patients were enrolled into CART-SIE study and leukapheresed; 85% (503) of them were infused and 83% (489), with a minimum follow-up of 30 days, were analyzed; 229 received tisa-cel and 260 axi-cel. (Fig. 1).

The present study analyzed the outcome of the 260 patients treated with axi-cel: 27% (70/260) had a diagnosis of PMBCL, 73% (190) of other LBCL, including 112 DLBCL-NOS, 44 HGBCL and 34 tFL.

As expected, when comparing PMBCL versus other LBCL, some clinical differences emerged in terms of age [median 33 years (IQR 27, 42) versus 57 years (IQR 50, 65), *p* < 0.0001], sex (male 51% versus 66%, *p* = 0.0312), refractory disease (90% versus 74%, *p* = 0.0048), advanced stage III–IV (39% versus 66%, *p* = 0.0001), intermediate/high or high International Prognostic Index (IPI) (21% versus 39%, *p* = 0.0078), bulky disease (57% versus 35%, *p* = 0.0015) (Table 1).

**Table 1:** Clinical characteristics.

	PMBCL (*N* = 70)	Other LBCL (*N* = 190)	*p* value
Sex			0.0312
Female	34 (49%)	64 (34%)	
Male	36 (51%)	126 (66%)	
Age			0.0001
Mean (SD)	35.0 (11.4)	55.9 (12.3)	
Median [Q1, Q3]	33.0 [27.0, 41.8]	57.5 [50.0, 65.0]	
Disease status			0.0048
Refractory	63 (90.0%)	141 (74%)	
Relapse	6 (9%)	46 (22%)	
Missing	1 (1%)	3 (2%)	
Ann Arbor			0.0001
I–II	43 (61%)	64 (34%)	
III–IV	27 (39%)	126 (66%)	
LDH			0.0958
Mean (SD)	255 (243)	344 (574)	
Median [Q1, Q3]	190 [154, 264]	203 [167, 314]	
Missing	4 (6%)	8 (4%)	
IPI			0.0078
<3	55 (79%)	113 (59%)	
≥3	15 (21%)	74 (39%)	
Missing	0	3 (2%)	
Bulky disease			0.0015
No	29 (42%)	122 (64%)	
Yes	40 (57%)	66 (35%)	
Missing	1 (1%)	2 (1%)	
Number of previous treatments			0.3055
Mean (SD)	2.60 (0.858)	2.49 (0.832)	
Median [Q1, Q3]	2 [2, 3]	2 [2, 3]	
Missing	0	3 (2%)	
Previous ASCT			0.0778
No	58 (83%)	136 (72%)	
Yes	12 (17%)	54 (28%)	

*PMBCL* primary mediastinal B-cell lymphoma, *other* *LBCL* large B-cell lymphoma other than primary mediastinal B-cell lymphoma, *SD* standard deviation, *Q1* interquartile 1, *Q3* interquartile 3, *LDH* lactate dehydrogenase, *IPI* international prognostic index, *ASCT* autologous stem cell transplantation.

### Bridging therapy

Bridging therapy was delivered to 83% (58/70) PMBCL patients and to 82% (155/190) other LBCL (*p* = 0.5782). The bridging therapies administered in PMBCL versus other LBCL were as follows: chemoimmunotherapy in 56% (39/70) versus 61% (115/190); radiotherapy in 21% (15) versus 15% (28); combined modality (chemoimmunotherapy plus radiotherapy) in 6% (4) versus 6% (12), respectively. Of note, 34% (88/260) patients received novel drugs for bridging, 41% (29/70) PMBCLs and 31% (59/190) other LBCLs; in details: 11% (29/260) were exposed to CPIs, 40% (28/70) PMBCL (16 received nivolumab in combination with brentuximab-vedotin, 2 single agent nivolumab and 10 pembrolizumab), and one other LBCL (pembrolizumab in combination with radiotherapy); 16% (41/260) to polatuzumab-vedotin containing regimens, one PMBCL and 21% (40/190) other LBCLs; 6% (15/260) to lenalidomide, one PMBCL and 5% (10/190) other LBCLs. Anti CD20 × CD3 bispecific antibody was used in two and ibrutinib in one other LBCL.

For PMBCL, the ORR and CR after bridging were 41% (29/70) and 17% (12/70), respectively; for other LBCL, the ORR and CR rate were 28% (53/190) and 6% (11/190), respectively, *p* = 0.0102. Patients who achieved a disease response after bridging had a better outcome compared to those not responding, with 12-month PFS of 57% (95% CI: 45–71) versus 45% (95% CI: 36–56), *p* 0.0425, and 12-month OS of 80% (95% CI: 69–92) versus 65% (95% CI: 58–76), *p* 0.0027.

### Safety

All the 260 patients were evaluable for safety at the time of the analysis (Table 2). All grades CRS was reported in 88% (228/260) of the patients, 86% (60/70) PMBCL and 88% (168/190) other LBCL, *p* = 0.5310; grade 3–4 CRS was registered in 13% (9/70) PMBCL and in 7% (14/190) other LBCL; one LBCL patient had a grade 5 CRS.

**Table 2:** Toxicities.

	PMBCL (*N* = 70)	Other LBCL (*N* = 190)	Overall (*N* = 260)	*p* value
CRS				0.5310
No	10 (14%)	22 (12%)	32 (12%)	
Yes	60 (86%)	168 (88%)	228 (88%)	
Grade 1	29 (41%)	95 (50%)	124 (48%)	
Grade 2	22 (31%)	58 (30%)	80 (31%)	
Grade 3	7 (10%)	13 (7%)	20 (8%)	
Grade 4	2 (3%)	1 (<1%)	4 (1%)	
Grade 5	0	1 (<1%)	1 (<1%)	
ICANS				0.3758
No	43 (61%)	129 (68%)	172 (66%)	
Yes	27 (39%)	61 (32%)	88 (34%)	
Grade 1	8 (11%)	23 (12%)	31 (12%)	
Grade 2	5 (7%)	20 (10%)	25 (10%)	
Grade 3	6 (9%)	13 (7%)	19 (7%)	
Grade 4	4 (6%)	5 (3%)	12 (5%)	
Grade 5	4 (6%)	0	4 (1%)	
Tocilizumab				0.9999
No	19 (27%)	52 (27%)	71 (27%)	
Yes	51 (73%)	138 (73%)	189 (73%)	
Steroids				0.5471
No	46 (66%)	133 (70%)	179 (69%)	
Yes	24 (34%)	57 (30%)	81 (31%)	
ICU admission				0.0148
No	54 (77%)	170 (90%)	224 (86%)	
Yes	16 (23%)	20 (10%)	36 (14%)	
Other AEs				0.8349
No	8 (11%)	25 (13%)	33 (13%)	
Yes	62 (89%)	165 (87%)	227 (87%)	
Anemia				0.0236
No	30 (43%)	52 (27%)	82 (32%)	
Yes	40 (57%)	138 (73%)	178 (68%)	
Grade 1–2	28 (40%)	95 (50%)	123 (47%)	
Grade 3	9 (13%)	36 (19%)	45 (17%)	
Grade 4	3 (6%)	7 (4%)	10 (4%)	
Leucopenia				0.6310
No	19 (27%)	46 (24%)	65 (25%)	
Yes	51 (73%)	144 (76%)	195 (75%)	
Grade 1–2	7 (10%)	20 (10%)	27 (10%)	
Grade 3	15 (21%)	36 (19%)	51 (20%)	
Grade 4	29 (41%)	88 (46%)	117 (45%)	
Neutropenia				0.7242
No	12 (17%)	37 (20%)	49 (19%)	
Yes	58 (83%)	153 (80%)	211 (81%)	
Grade 1–2	6 (9%)	17 (9%)	23 (9%)	
Grade 3	7 (10%)	19 (10%)	26 (10%)	
Grade 4	45 (64%)	117 (62%)	162 (62%)	
Thrombocytopenia				0.2532
No	31 (44%)	69 (36%)	100 (39%)	
Yes	39 (56%)	121 (64%)	160 (61%)	
Grade 1–2	23 (33%)	47 (25%)	70 (27%)	
Grade 3	7 (10%)	32 (17%)	39 (15%)	
Grade 4	9 (13%)	42 (22%)	51 (20%)	
Febrile neutropenia				0.0984
No	54 (77%)	126 (66%)	180 (69%)	
Yes	16 (23%)	64 (34%)	80 (31%)	
Grade 1–2	4 (6%)	12 (6%)	16 (6%)	
Grade 3	7 (10%)	38 (20%)	45 (17%)	
Grade 4	5 (7%)	14 (7%)	19 (7%)	
Cardiac				0.2416
No	64 (91%)	181 (95%)	245 (94%)	
Yes	6 (9%)	9 (5%)	15 (6%)	
Grade 1–2	2 (3%)	7 (4%)	9 (3%)	
Grade 3	3 (4%)	2 (2%)	5 (2%)	
Grade 4	1 (1%)	0	1 (<1%)	

*PMBCL* primary mediastinal B-cell lymphoma, *other LBCL* large B-cell lymphoma other than primary mediastinal B-cell lymphoma, *CRS* cytokine release syndrome, *ICANS* immune-effector cell associated neurotoxicity syndrome, *ICU* intensive care unit, *AEs* adverse events.

All grades ICANS was reported in 34% (88/260) of the patients, 39% (27/70) PMBCL and 32% (61/190) LBCL, *p* = 0.3758; grade 3–4 ICANS was registered in 19% (13/70) PMBCL and in 9% (18/190) LBCL; 6% (4/70) PMBCL had a grade 5 ICANS. Regarding the four PMBCLs who developed a fatal neurological event, none of them had a lymphoma dissemination at central nervous system nor at diagnosis nor at the time of axi-cel infusion; three of them were exposed to pembrolizumab as bridging therapy, with a wash-out period of at least four weeks before starting lymphodepletion for CAR-T cells therapy. All of them received levetiracetam as primary prophylaxis of neurological events, and, at the time of the event, received high dose steroids, tocilizumab and anakinra according to the risk management plan of the product and to national recommendations. Imaging was performed in all the cases, showing cerebral edema in one, and areas of inflammatory tissue distress in the other three patients. Only in one case was performed a diagnostic lumbar puncture, with no pathological results. No autopsy was performed. The timing of death was, respectively, 4 days, 5 days, 15 days after axi-cel infusion for the first three patients; the last one, with unresolved grade 4 ICANS, died 2 months after infusion due to an acute encephalomyelitis. The CAR-T expansion in blood demonstrated an expansion of 83 and 145 cells per microliters at day 7 after infusion in the two patients alive at that timepoint.

Tocilizumab and steroids were infused in 83% and 35% of the patients that experienced CRS, respectively and were equally distributed between PMBCL and other LBCL, *p* = 0.9999 and *p* = 0.5471.

NRM at 30 and 90 days after infusion were 2.7% (95% CI: 1.3–5.6%) and 3.1% (95% CI: 1.6–6.2%), respectively: 4 PMBCLs due to grade 5 ICANS and 4 other LBCLs, one due to grade 5 CRS, 3 to gram negative septic shock.

At 30-day after infusion, all grade hematological and non-hematological toxicities were observed in 87% (227/260) of the patients, 89% (62/70) in PMBCL and 87% (165/190) in other LBCL, *p* = 0.8349; the most frequent were hematological: grade 3–4 neutropenia in 72% (188/260), grade 3–4 thrombocytopenia in 35% (90/260), grade 3–4 anemia in 21% (55/260). Febrile neutropenia was recorded in 31% (80/260) of the patients, 23% (16/70) PMBCL versus 34% (64/190), *p* = 0.0984.

### Disease response and survival outcomes

Median follow-up time for the 260 infused patients was 12.17 months (IQR 5.53, 22.73). At 30-day, disease response was evaluable in 255 patients (68 PMBCLs and 187 other LBCLs); the response was not evaluable in five patients (imaging not performed) and in seven patients who died for adverse events. ORR was 78% (53/68) with 50% (34) CR and 28% (19) PR in PMBCLs, and 75% (141/187) with 53% (100) CR and 22% (41) PR in other LBCLs; stable disease (SD) was observed in 10% (7/68) PMBCLs and in 10% (18/187) other LBCLs; 7% (5/68) PMBCLs and 13% (24/187) other LBCLs experienced progressive disease (PD), *p* = 0.5457.

The response at 90-day after the infusion was evaluable in 227 patients (65 PMBCLs and 162 other LBCLs); the response was not evaluable in 25 patients due to time-point not yet reached, and in one patient who died for toxicity at 90-day. ORR was 69% (45/65) with 65% (42/65) CR and 4% (3/65) PR in PMBCLs, and 54% (87/162), with 47% (76/162) CR and 7% (11/162) PR in other LBCLs; SD was observed in 3% (2/65) PMBCLs and in 4% (6/162) other LBCLs; 21% (14/65) PMBCLs and 40% (65/162) other LBCLs experienced PD, *p* = 0.0336.

The median DoR was not reached in PMBCLs and was 23.64 months in other LBCLs. The rate of patients still in response at 12-month was 74% (95% CI: 62–88) for PMBCLs and 56% (95% CI: 48–67) for other LBCL patients (*p* = 0.0147).

### Management of relapses after CAR T cells

Disease recurrence within the first month after the infusion was observed in 4% (3/70) of PMBCLs and 11% (21/190) of other LBCLs (*p* = 0.4384); 10% (7/70) of PMBCL patients and 11% (21/190) other LBCLs progressed after PR; 6% (4/70) of PMBCLs and 14% (27/190) of other LBCLs relapsed. Salvage treatment for PD or relapse was performed in 20% (14/70) of PMBCLs and in 25% (47/190) of other LBCLs; among PMBCLs: 57% (8/14) received CPIs and 21% (3/14) received bispecific antibodies; among other LBCLs: 41% (11/27) were treated with bispecifics antibodies, 26% (7/27) with polatuzumab-vedotin containing regimens and 22% (6/27) with lenalidomide at relapse.

**Fig. 2:**
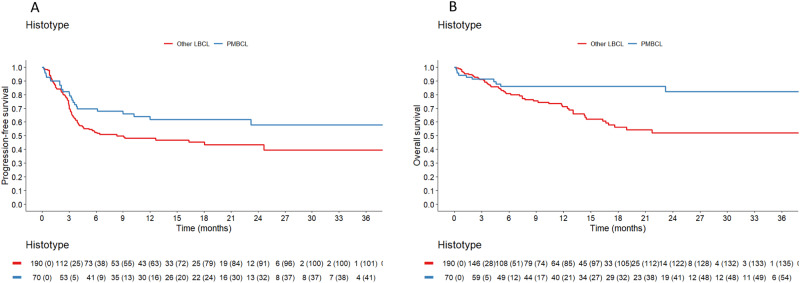
Progression-free survival and Overall survival. **A** Progression-free survival. PMBCL primary mediastinal B-cell lymphoma, other LBCL large B-cell lymphoma other than primary mediastinal B-cell lymphoma. Log-rank test *p* value 0.0386. **B** Overall survival. PMBCL primary mediastinal B-cell lymphoma, other LBCL large B-cell lymphoma other than primary mediastinal B-cell lymphoma. Log-rank test *p* value 0.0034.

The 12-month PFS was 62% (95% CI: 51–75) in PMBCL versus 48% (95% CI: 41–57) in other LBCL (log-rank p 0.0386) (Fig. 2A). Considering the different subtypes in other LBCL, the 12-month PFS was 41% (95% CI: 32–52) in DLBCL-NOS, 56% (95% CI: 42–76) in HGBCL, 67% (95% CI: 52–88) in tFL (log-rank *p* 0.0389); considering the 12-month PFS between DLBCL-NOS and PMBCL, the log-rank was *p* 0.0128 (Fig. S1A).

The 12-month OS was 86% (95% CI: 78–95) in PMBCL versus 71% (95% CI: 64–79) in other LBCL (log-rank *p* 0.0034) (Fig. 2B). Considering the different subtypes in other LBCL, the 12-month OS was 74% (95% CI: 65–84) in DLBCL-NOS, 62% (95% CI: 47–82) in HGBCL and 73% (95% CI: 55–95) in tFL (log-rank *p* 0.0058); considering the 12-month OS between DLBCL-NOS and PMBCL, the log-rank was *p* 0.016 (Fig. S1B; the OS from the time of relapse between PMBCL and other LBCL is reported in Fig. S2).

Among patients treated with axi-cel, PMBCL had a significantly better PFS and OS than other LBCL in an univariable Cox analysis and the better prognosis of PMBCL compared to other LBCL was maintained in a multivariable Cox analysis performed to adjust the comparison for clinically relevant variables (age, relapsed or refractory disease status, Ann Arbor stage, international prognostic index, bulky disease, previous auto-SCT, bridging therapy) (Table 3).

**Table 3:** Univariate and multivariate Cox model.

Variable	HR	Lower 0.95	Upper 0.95	*p* value
**Univariable Cox model for PFS**				
Histology PMBCL vs. other LBCL	0.62	0.40	0.98	0.0387
**Multivariable Cox model for PFS**				
Histology PMBCL vs. other LBCL	0.59	0.34	1.04	0.0683
Age 63 vs. 39	1.08	0.71	1.62	0.7282
Disease status relapse vs. refractory	0.74	0.43	1.25	0.2577
Ann Arbor III–IV vs. I–II	1.56	1.01	2.43	0.0473
IPI ≥ 3 vs. < 3	1.26	0.81	1.94	0.3056
Bulky disease yes vs. no	1.75	1.16	2.64	0.0074
ASCT yes vs. no	0.60	0.37	0.99	0.0463
Bridging therapy yes vs. no	0.85	0.52	1.39	0.5122
**Univariable Cox model for OS**				
Histology PMBCL vs. other LBCL	0.38	0.19	0.75	0.0051
**Multivariable Cox model for OS**				
Histology PMBCL vs. other LBCL	0.39	0.17	0.88	0.0227
Age 63 vs. 39	1.15	0.67	1.99	0.6044
Disease status relapse vs. refractory	0.81	0.41	1.59	0.5375
Ann Arbor III–IV vs. I–II	3.00	1.51	5.98	0.0018
IPI ≥ 3 vs. < 3	1.20	0.68	2.10	0.5249
Bulky disease yes vs. no	2.34	1.37	3.98	0.0018
ASCT yes vs. no	0.77	0.42	1.42	0.3975
Bridging therapy yes vs. no	1.62	0.71	3.69	0.2543

*PFS* progression-free survival, *PMBCL* primary mediastinal B-cell lymphoma, *other LBCL* large B-cell lymphoma other than primary mediastinal B-cell lymphoma, *HR* hazard ratio, *IPI* international prognostic index, *ASCT* autologous stem cell transplantation, *OS* overall survival.

## Discussion

At present, CART-SIE is the first large real-life prospective, multicenter, observational study evaluating the outcome of patients affected by PMBCL and other LBCL. In our series, 14% of patients treated with CAR-T cells were PMBCL; the relatively higher than expected number of PMBCL, has not a clear explanation since all cases were consecutively enrolled. At a median follow-up time of 12.17 months, PMBCL had a superior DoR, PFS and OS when compared to other LBCL. Our real-life results are superimposable to those reported by the ZUMA-1 trial in a smaller cohort of patients. However, it should be emphasized that in our study, four PMBCL patients died for neurologic adverse events in the first 2 months after axi-cel infusion, and three of them were previously exposed to CPIs. Due to the small number of the events, and due to heterogeneity of the clinical characteristics of the four patients, it is not possible to perform a formal analysis comparing them to the overall population. We are currently unable to determine whether the use of CPIs increases axi-cel neurological toxicities, but this is a clinical alert that should be kept into consideration. Notably, the clinical spectrum of neurological events related to CPIs reported in literature is varied, and includes myositis, myasthenic syndromes, peripheral neuropathies, encephalitis, and may be severe and life-threatening [24].

In the Zuma-1 pivotal study, only 8 patients with R/R PMBCL were enrolled into the trial and treated with axi-cel, and also in the subsequent real-world experiences, a small proportion of PMBCL was treated, and no disaggregated data were reported [11, 14, 15]. In the Transcend NHL 001 study, 15 patients with R/R PMBCL were enrolled; compared to other subtypes, PMBCL and tFL had longer DoR and PFS [13]. The ORR, PFS and OS of the 70 PMBCL enrolled in our study, were similar to the retrospective study reported by Crombie et al. in 33 R/R PMBCL [16]. In their analysis, the authors explored the sequencing of axi-cel with CPIs, and the impact of the combination on outcome; with the limitation of the small numbers, authors concluded that there is no evidence of a beneficial impact adding CPI in a CAR-T program. In our experience, 34% (24/70) PMBCL patients received CPIs as bridging therapy, and half of them achieved a response that was maintained after CAR-T infusion.

In our study, the bridging therapy was performed in more than 80% of the patients; as reported by Bethge et al., also in our hand, the bridging success was a critical determinant of CAR T-cell therapy outcome [25]. On these bases, PFS and OS at the end of treatment could be influenced by the response to bridging, making the goal of obtaining the lowest tumor burden at the time of CAR T-cell infusion an attractive strategy [26]. In our series, response to bridging was observed in 31% (82/260), with a superior ORR in PMBCL (41%) compared to other LBCL (28%). We postulate that this advantage may be related to the use of CPIs.

We observed that the CR rate at 30 days was similar in PMBCLs and other LBCL, but at 90 days was superior in PMBCLs (65%) compared to other LBCLs (47%), and accordingly the rate of progression at 90-day after infusion is inferior in PMBCLs (21%) compared to other LBCLs (40%). Those results translated into a median DoR not reached in PMBCLs compared to median DoR of 23.64 months in other LBCLs. A possible explanation is that the CR “quality” in PMBCLs seems superior compared to other LBCLs, with less relapses, and this translated into better PFS and OS. Whether the benefit is related to the intrinsic biology of PMBCLs or to the use of CPIs during bridging therapy or both is impossible to determine at the present time, but warrants further investigation.

At relapse after CAR-T, in our study half of the R/R PMBCL were treated with CPIs as salvage treatment, and the majority of the R/R other LBCL received CD20 × CD3 T-cell engaging bispecific monoclonal antibodies or polatuzumab-vedotin or immunomodulatory drugs. The CPIs showed impressive results in R/R PMBCL [6, 8], as well as the bispecific antibodies demonstrated to be effective in R/R other LBCL patients, even in CAR-T failures [26–28]; these novel drugs represent a valuable opportunity in our patients at poor prognosis and further studies are needed to establish the best treatment sequencing. Furthermore, the sequencing of salvage therapies in PMBCL will need to be reviewed in light of the recent approval of axi-cel and liso-cel in patients with other LBCL refractory to first line treatment; however, patients with PMBCL were excluded from the ZUMA-7 study, and in the TRANSFORM study only 8 patients with PMBCLs were treated with liso-cel compared to 9 treated with standard of care [29, 30].

In conclusion, our study has important clinical implications with several novel insights: 1. axi-cel is a very effective salvage strategy for PMBCL, indicating CAR-T should not be deferred; 2. a CPI based bridging is feasible and able to improve the prognosis of PMBCL; 3. CRS is manageable and does not seem to increase after CPIs, however, it should be emphasized that neurologic adverse events must be strictly monitored.

### Supplementary information

Supplemental material

## Data Availability

The dataset analyzed during the current study is available from the corresponding author on reasonable request.
