# Supplementary material for: Axicabtagene ciloleucel treatment is more effective in primary mediastinal large B-cell lymphomas than in diffuse large B-cell lymphomas: the Italian CART-SIE study
Source: Leukemia. 2024 Mar 8;38(5):1107–14. doi: 10.1038/s41375-024-02213-x (PMC11073993; doi:10.1038/s41375-024-02213-x)

**Supplementary Appendix**

**Table of contents:**

1. Title
2. Patient selection criteria in accordance with Italian drug agency “AIFA”
   1. Inclusion criteria
   2. Exclusion criteria
3. Figure S1A
4. Figure S1B
5. Figure S2
6. **Title.**

Axicabtagene ciloleucel treatment is more effective in primary mediastinal large B-cell lymphomas than in diffuse large B-cell lymphomas: the Italian CART-SIE study

1. **PATIENT SELECTION CRITERIA**

Eligible patients were relapsed/refractory patients affected by aggressive large B-cell lymphomas, including diffuse large B-cell lymphomas (DLBCL) [DLBCL not otherwise specified (DLBCL-NOS), high-grade B-cell lymphoma (HGBCL), DLBCL arising from transformed follicular lymphoma (tFL)] and PMBCL, after at least two treatment lines.

**2.1. Inclusion criteria**

1. age ≥18 e ≤ 75 years

2. Refractory DLBCL; relapsed DLBCL; PMBCL.

3. Ann Arbor (Lugano modified) Stage I, IE, II, IIE, II bulky, III, IV

4. International Prognostic Index 0, 1, 2, 3, 4, 5.

5. Eastern Cooperative Oncology Group Performance status: 0; 1.

6. Life expectation > 12 weeks

7. Number of prior treatments (including rituximab and anthracyclines): 1, 2, 3, > 4.

8. Relapse after autologous stem cell transplantation: yes, no.

9. Patient eligible to autologous stem cell transplantation: no.

10. Previous allogenic stem cell transplantation: no; if yes, no acute or chronic graft versus host disease syndrome, no immunosuppressive therapies during the last six months, interval from transplant of at least 6 months.

11. Previous anti-CD19 therapy (including out of specification chimeric antigen receptor (CAR) T-cell therapies or other therapies): no; if yes, confirmed CD19 expression.

12. HIV negativity and absence of active hepatitis B virus (HBV) or hepatitis C virus (HCV) infection.

13. No active Central Nervous System (CNS) disease.

14. Absence of signs of intracranial hypertension.

15. Absence of seizures in the last three months.

16. Absence of autoimmune disease requiring immunosuppressive therapies.

17. Clearance of creatinine > 60 ml/min.

18. Levels of serum transaminases < 2.5 the upper normal limit, and bilirubine < 1.5 the upper normal level (< 3 in Gilbert syndrome)

19. Ejection fraction > 50% and no myocardial stroke or relevant abnormalities at electrocardiogram.

20. Adequate lung function (dyspnea < 1, oxygen saturation > 92%, absence of pericardial effusion).

21. Adequate bone marrow reserve: neutrophils > 1000/mm^3^, lymphocytes > 100/mm^3^, platelets > 75/mm^3^, hemoglobin > 8 g/dl.

22. Absence of deep vein thrombosis or pulmonary embolism in the last six months.

23. Signed informed consent.

**2.2. Exclusion criteria**

1. age <18 e > 75 years

2. Primary central nervous system B-cell lymphoma; Richter syndrome; hystology other than: DLBCL; PMBCL.

3. Eastern Cooperative Oncology Group Performance status: 2; 3; 4.

4. Life expectation < 12 weeks

5. Patient eligible to autologous stem cell transplantation: yes.

6. Previous allogenic stem cell transplantation: if yes, with acute or chronic graft versus host disease syndrome, or immunosuppressive therapies during the last six months, or interval from transplant less than 6 months

7. Previous anti-CD19 chimeric antigen receptor (CAR) T-cell therapies and/or previous anti-CD19 therapy without CD19 expression.

8. Active Central Nervous System (CNS) disease.

9. Signs of intracranial hypertension.

10. Seizures in the last 3 months.

11. Autoimmune disease requiring immunosuppressive therapies in the last two years.

12. Clearance of creatinine < 60 ml/min.

13. Levels of serum transaminases > 2.5 the upper normal limit, and bilirubine > 1.5 the upper normal level (> 3 in Gilbert syndrome)

14. Ejection fraction < 50% and no myocardial stroke or relevant abnormalities at electrocardiogram.

15. Insufficient lung function (dyspnea > 1, oxygen saturation < 92%, presence of pericardial effusion).

16. Not adequate bone marrow reserve: neutrophils < 1000/mm^3^, lymphocytes < 100/mm^3^, platelets < 75/mm^3^, hemoglobin < 8 g/dl.

17. Deep vein thrombosis or pulmonary embolism in the last six months.

1. **Figure S1A. Progression-free survival by histological subgroups.**

Figure legend.

DLBCL: diffuse large B-cell lymphoma; HGBCL: high-grade B-cell lymphoma; tFL: transformed follicular lymphoma; PMBCL: primary mediastinal B-cell lymphoma.

Log-rank test p-value: 0.0389; Log-rank test (PMBCL vs DLBCL) p-value: 0.0128.


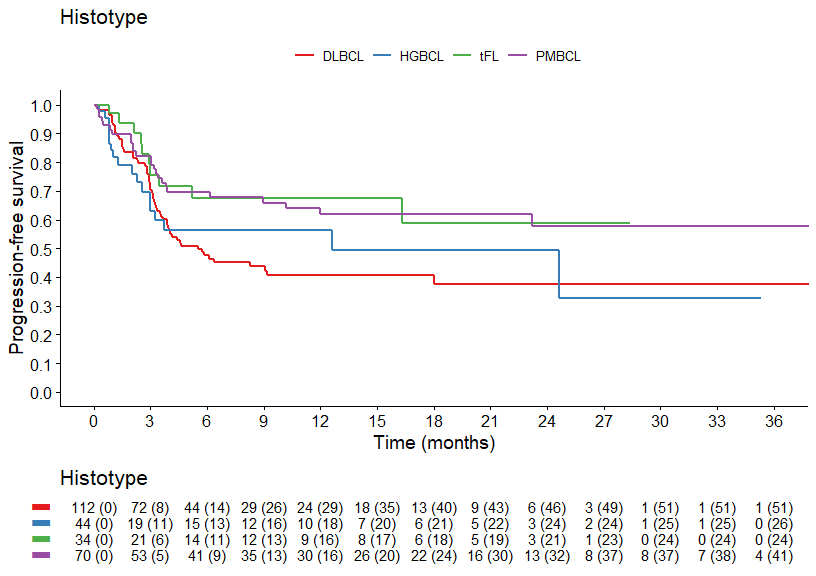


1. **Figure S1B. Overall survival by histological subgroups.**

Figure legend.

DLBCL: diffuse large B-cell lymphoma; HGBCL: high-grade B-cell lymphoma; tFL: transformed follicular lymphoma; PMBCL: primary mediastinal B-cell lymphoma.

Log-rank test p-value: 0.0058; Log-rank test (PMBCL vs DLBCL) p-value: 0.016.


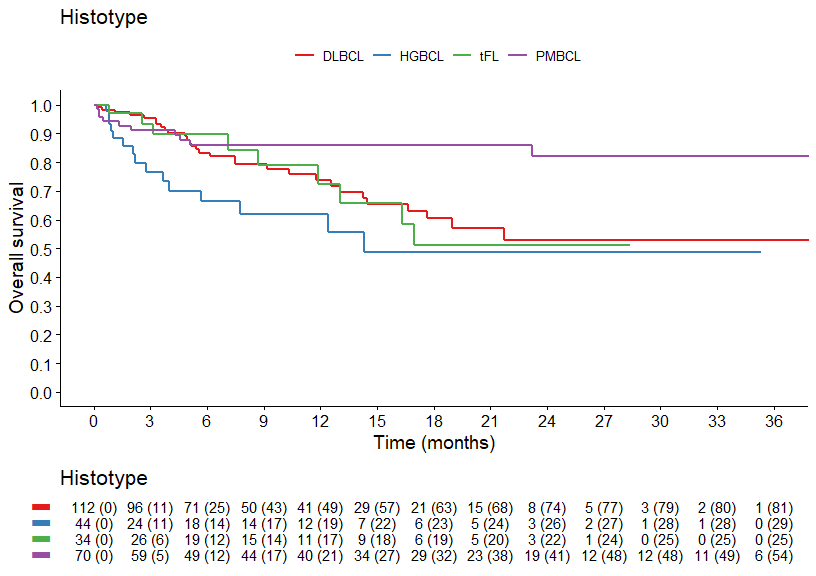


1. **Figure S2. Overall survival from the time of relapse.**

Figure legend.

PMBCL: primary mediastinal B-cell lymphoma; other LBCL: large B-cell lymphoma other than primary mediastinal B-cell lymphoma.

Log-rank test p-value: 0.0041.


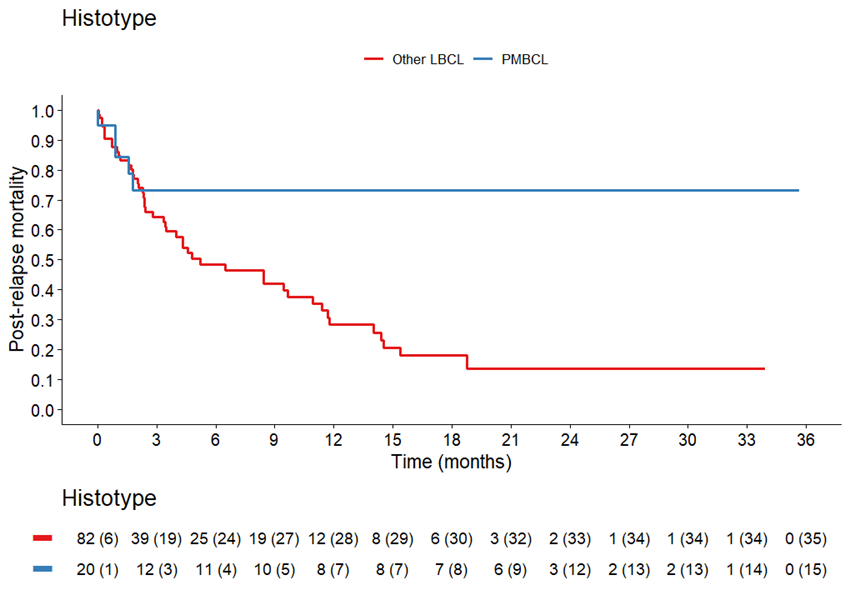

Supplement: Supplementary file 1 — Supplemental material [file 41375_2024_2213_MOESM1_ESM.docx]
